# Effect of Ag/Al co-doping method on optically p-type ZnO nanowires synthesized by hot-walled pulsed laser deposition

**DOI:** 10.1186/1556-276X-7-273

**Published:** 2012-05-30

**Authors:** Kyoungwon Kim, Deuk-Hee Lee, Sang Yeol Lee, Gun-Eik Jang, Jin-Sang Kim

**Affiliations:** 1 Electronic Materials Research Center, Korea Institute of Science and Technology, P.O. Box 131, Cheongryang, Seoul, 130-650, South Korea; 2 Department of Semiconductor Engineering, Cheongju University, Cheongju, Chungbuk, 360-764, South Korea; 3 Department of Advanced Materials Engineering, Chungbuk National University, Cheongju, 361-763, South Korea; 4 Department of Materials Science and Engineering, University of Michigan-Ann Arbor, Ann Arbor, MI, 48109, USA

**Keywords:** SAZO nanowire, Photoluminescence, Ag/Al co-doping method, Exciton bound to a neutral acceptor

## Abstract

Silver and aluminum-co-doped zinc oxide (SAZO) nanowires (NWs) of 1, 3, and 5 at.% were grown on sapphire substrates. Low-temperature photoluminescence (PL) was studied experimentally to investigate the p-type behavior observed by the exciton bound to a neutral acceptor (A^0^X). The A^0^X was not observed in the 1 at.% SAZO NWs by low-temperature PL because 1 at.% SAZO NWs do not have a Ag-O chemical bonding as confirmed by XPS measurement. The activation energies (*E*_a_) of the A^0^X were calculated to be about 18.14 and 19.77 meV for 3 and 5 at.% SAZO NWs, respectively, which are lower than the activation energy of single Ag-doped NW which is about 25 meV. These results indicate that Ag/Al co-doping method is a good candidate to make optically p-type ZnO NWs.

## Background

Zinc oxide (ZnO) has emerged as a superior n-type II-VI compound semiconductor material with high chemical and physical stability as well as a direct, wide bandgap (3.37 eV) and high exciton binding energy (60 meV) at room temperature [1]. ZnO has potential applications due to its various attractive properties such as transparency, ferromagnetic, optical, photoelectric, gas sensing, and piezoelectric properties and a wide range of electrical properties, from dielectric to conductive materials [2-5]. Wide-bandgap ZnO optoelectronics have been stimulated by the investigation of materials for use in the next generation of short-wavelength optoelectronic devices and have revealed good results, including high transmittance and mobility [6,7]. As-grown ZnO nanostructures typically show an n-type semiconductor due to their intrinsic defects such as oxygen vacancies and zinc interstitials [8]. The strong n-type conductivity of ZnO restricts its application, which makes it difficult to fabricate p-type ZnO materials [5], and the realization of p-type ZnO is rather difficult due to its asymmetric doping limitations [9]. Recently, research on ZnO has been focused on the synthesis of p-type ZnO using various dopants including N, P, As, Sb, and Ag [5,10-12]. Among possible acceptor dopants, Ag (a group Ib element) is a good candidate for producing a shallow acceptor level in ZnO, if incorporated on substituted Zn sites [13]. The Ag-doped ZnO for the various applications has been reported by Kang et al. [14]. They demonstrated that the Ag ion can be substituted into the site of the Zn ion and a narrow window region exists to fabricate the p-type ZnO using Ag as a p-type dopant source. However, there have been no reports on the perfect fabrication of p-type ZnO nanowires (NWs) using Ag dopant.

Several research groups proposed a co-doping method where the acceptor dopants and group III elements are used such as co-doping of nitrogen and gallium by pulsed laser deposition (PLD), nitrogen and indium by ultrasonic spray pyrolysis, and nitrogen and aluminum by reactive magnetron sputtering [15-17]. Lu et al. reported to fabricate p-type ZnO structures by co-doping with nitrogen and aluminum [17]. They demonstrated that simultaneous co-doping using acceptors and reactive donors could be expected to enhance the solubility of acceptors in ZnO and to raise shallow acceptor levels in the bandgap [17,18]. Therefore, the incorporation of acceptors in ZnO was remarkably enhanced due to the presence of aluminum. However, controlling nitrogen at room temperature is very difficult because nitrogen in air exists in a gas state. So, we used Ag dopant for making a p-type semiconductor in a ZnO matrix instead of nitrogen.

In this work, we have investigated the growth behavior of various silver and aluminum-co-doped zinc oxide (SAZO) NWs on an Al_2_O_3_ (0001) substrate by hot-walled pulsed laser deposition (HW-PLD) which is one of the special methods for nanostructure synthesis. After optimizing the process condition for NW formation in the HW-PLD, we verified the Ag-doping status, confirmed the exciton bound to a neutral acceptor (A^0^X) by low-temperature (13 K) photoluminescence (PL), and compared thermal activation energies (*E*_a_) of single-doped and co-doped ZnO-based NWs.

## Methods

The fabrication methods of NWs have been developed to be realized at nanoscale such as thermal evaporation, PLD, and wet chemical processing [19,20]. Among various growth methods, the PLD has several notable advantages which are very effective in obtaining the stoichiometry of synthesized materials on the substrate as a target than many other gas surface-based growth techniques [21]. SAZO NWs of 1, 3, and 5 at.% have been synthesized on sapphire (0001) substrates in HW-PLD with a 20-Å Au film as a catalyst. SAZO NWs of 1, 3, and 5 at.% are grown in a furnace temperature of 800 °C with Ar gas of 90 sccm and a working pressure of 1.2 Torr. The HW-PLD has a target rotating system ensuring homogeneous target ablation. A KrF excimer laser (248 nm) operating at a pulse repetition rate of 10 Hz is focused onto pure ZnO and 1, 3, and 5 at.% SAZO targets for the deposition. The energy density of the laser is set to 1.2 J/cm^2^, and the shot area on the target surface is 0.042 cm^2^. Before synthesis, there should be a pre-deposition process with a laser shot time of 5 min on the surface of the target. The deposition process is continued for 30 min.

The target in this process is synthesized using high-purity ZnO (99.999%, Kojundo Chemical Laboratory, Sakado-shi, Japan), Ag_2_O (99.99%, Kojundo Chemical Laboratory), and Al_2_O_3_ (99.99%, Kojundo Chemical Laboratory) powders. The ethanol-based solutions of Ag and Al powders are ground and mixed with the ZnO powder by planetary milling for 48 h. The target is finalized by sintering at 950 °C for 3 h.

The low-temperature PL spectroscopy is a very sensitive tool for characterizing acceptor/donor impurities and is helpful to understand the optical and electrical performances of the materials. We focus on the temperature dependence of PL measurements of various SAZO NWs to reveal the role of the Ag acceptor in the optical properties of the ZnO NWs. Temperature-dependent PL (Dongwoo Fine-Chem Co., Ltd., Seoul, South Korea) spectra analyses are performed from 13K to room temperature using a 325-nm He-Cd laser, and a cycle refrigeration system is used to lower the temperature of the sample to 13 K during the low-temperature PL measurement. The crystal morphology is characterized using a field-emission scanning electron microscope (FE-SEM) and a transmission electron microscope (TEM), and the Ag element is observed in ZnO NWs by X-ray photoelectron spectroscopy (XPS) which is carried out to investigate the elemental composition of ZnO-based NWs.

## Results and discussions

**Figure 1:**
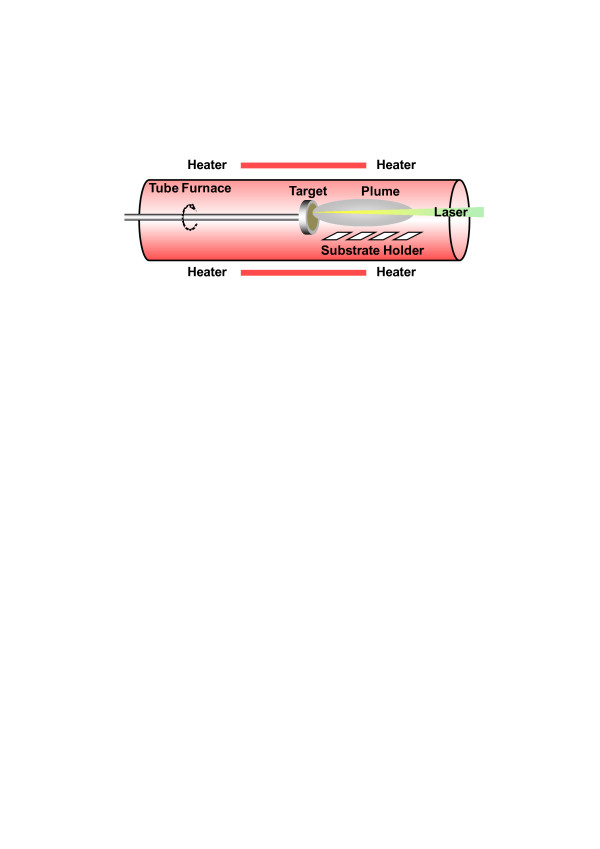
**A schematic diagram of the HW-PLD system for the fabrication of the various SAZO NWs.** The self-designed HW-PLD enables the synthesis of oxide NWs while controlling the doping concentration featuring doping control by adjusting the target composition since it guarantees the transfer of the composition from the target to the NWs.

**Figure 2:**
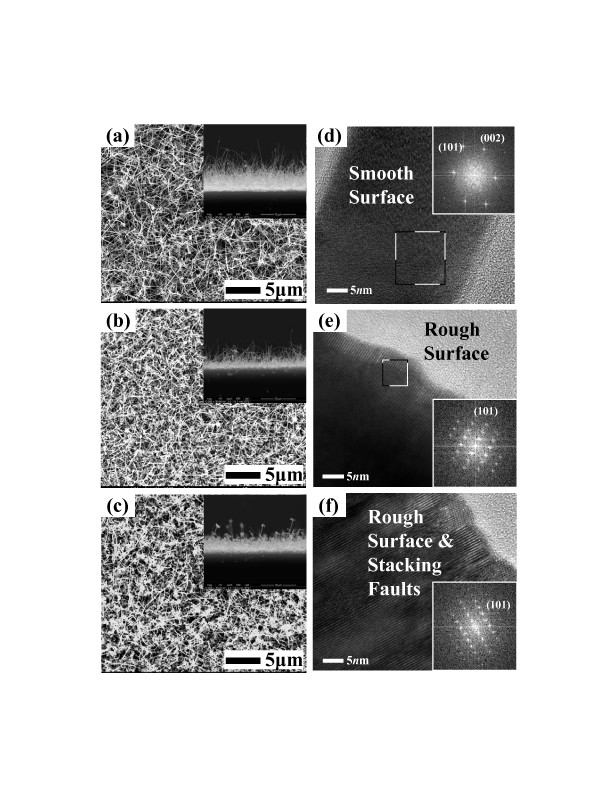
**The SEM and TEM images of various SAZO NWs.** The Ag/Al co-dopant concentration is set to (**a**, **d**) 1, (**b**, **e**) 3, and (**c**, **f**) 5 at.%, respectively. The insets are SAED patterns.

**Figure 3:**
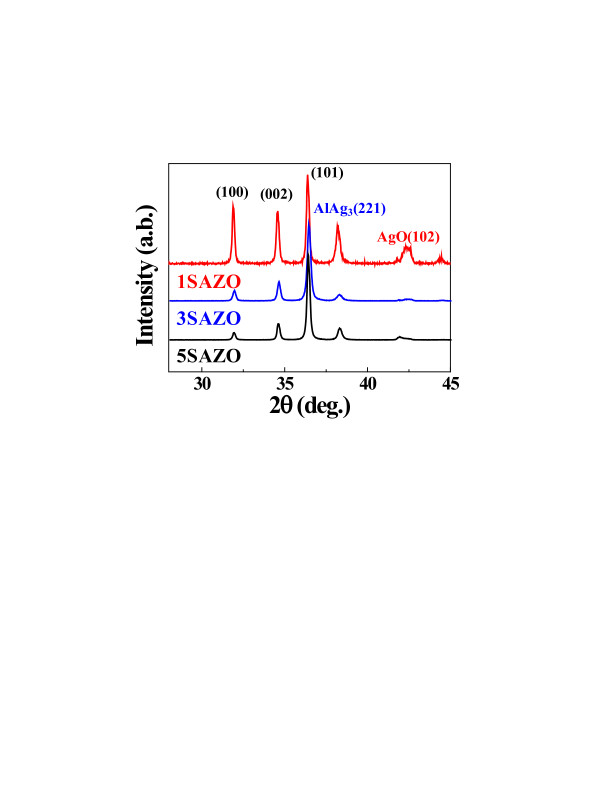
**The high-resolution XRD data.** All SAZO NWs reveal three peaks which originated from ZnO materials, including (100), (002), and (101).

**Figure 4:**
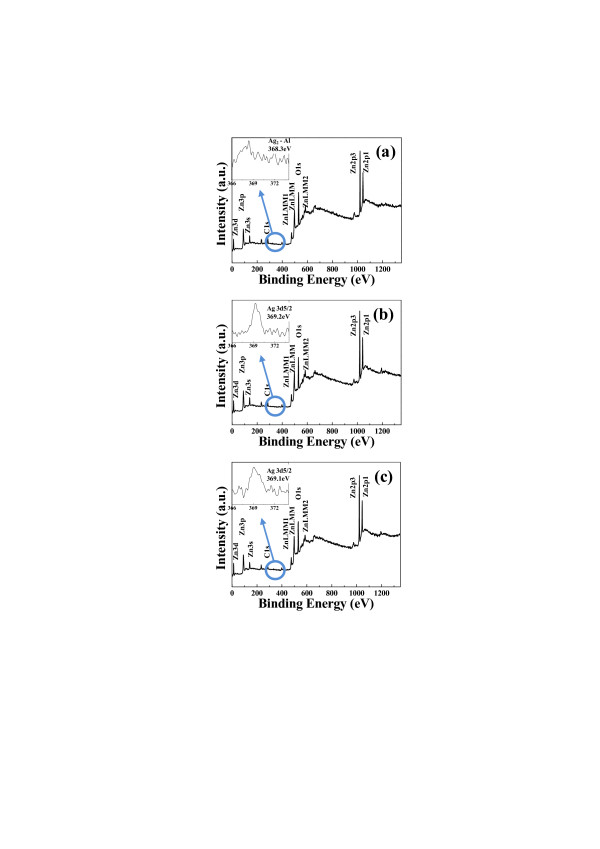
**The binding energy of both related Ag and Al elements by XPS measurement.** The binding energy of both related Ag and Al elements by XPS measurement for the presence of Ag dopant in (**a**) 1, (**b**) 3, and (**c**) 5 at.% SAZO NWs. A sharp, strong peak which originated from the Ag chemical bonding peak (Ag 3d5/2) of the SAZO NWs is observed at 369.2 and 369.1 eV for 3 and 5 at.% SAZO NWs, respectively. The doping conditions of 3 and 5 at.% SAZO NWs have been optimized to make Ag-doped ZnO NWs by Ag and Al co-doping technique.

Figure 1 shows a schematic diagram of the HW-PLD system for the fabrication of various SAZO NWs. The self-designed HW-PLD enables the synthesis of metal oxide NWs while controlling the doping concentration featuring doping control by adjusting the target composition since it guarantees the transfer of the composition from the target to the NWs. The KrF excimer laser beam enters along the tube furnace and is focused on the surface of Ag/Al-doped ZnO mixed target, thus allowing *in situ* modulation of chemical composition. The FE-SEM images show the controlled morphology of the SAZO NWs when the Ag/Al concentration is set to (a) 1, (b) 3, and (c) 5 at.%, respectively, as shown in Figure 2. The orientation and the distribution of the SAZO NWs with the diameter ranging from 50 to 90 nm and the length ranging from 3 to 7 μm are random, as shown in Figure 2. With the increasing Ag/Al co-doping concentrations, an irregular distribution of the SAZO NWs with different shapes has been observed. The diameter, length, and density of the SAZO NWs are clearly depending on the different doping concentrations. It is considered that the irregularity of the heavily doped sample stems from the lattice stress induced due to the substitution of Ag into the Zn site [22]. In order to investigate the effect of the Ag/Al co-doping method on structural properties of ZnO-based NWs, we have performed X-ray diffraction (XRD), high-resolution TEM, and selected area electron diffraction (SAED). Figure 2d,e,f shows HR-TEM images of 1, 3, and 5 at.% SAZO NWs, respectively. With the increasing Ag/Al doping concentration, the surface morphology of various SAZO NWs becomes rough, and stacking faults are generated. This means that the doping induces the stress which originated from the affinity mismatches and size between the original lattice elements and the substitutional dopants. The insets of Figure 2d,e,f are SAED patterns of 1, 3, and 5 at.% SAZO NWs, respectively. As shown in the inset of Figure 2d, the growth plane of 1 at.% SAZO NW shows two growth planes with (002) and (101). The (002) growth plane with a lattice spacing of about 0.26 nm is a *c*-axis of common wurtzite ZnO, and the (101) growth plane is another axis of common wurtzite ZnO [23]; the insets of Figure 2e,f show the primary growth plane which changed from (002) to (101) caused by Ag/Al co-doping stress which is another doping effect. Figure 3 shows high-resolution XRD data with 1, 3, and 5 at.% SAZO NWs. All SAZO NWs reveal three peaks which originated from ZnO materials, including (100), (002), and (101). However, both (100) and (002) peaks of 3 and 5 at.% SAZO NWs are decreased when the concentration of Ag/Al dopants increased over 3 at.%. This indicates that the primary growth direction of 3 and 5 at.% SAZO NWs is changed from (002) to (101). The primary growth (101) plane of SAZO NWs is attributed to the reduced doping stress in the ZnO lattice because the Ag^+^ ions have a larger radius (0.122 nm) compared with the host Zn^2+^ ions (0.072 nm). In the case of 1 at.% SAZO NW, it shows a (221) peak of Ag-Al chemical bonding located at 38.42 eV that is a defect as an interstitial dopant. Therefore, the XPS result of 1 at.% SAZO NW just shows Ag-Al chemical bonding at 368.3 eV as shown in Figure 4a. The energy-dispersive X-ray spectroscopy (EDX) spectra reveal the weight/atomic percent of Zn, Al, and Ag elements in various SAZO NWs as shown in Table 1. The atomic ratios of silver in 1, 3, and 5 at.% SAZO NWs are 0.17%, 1.02%, and 1.25%, respectively, and the weigh ratios of silver in 1, 3, and 5 at.% SAZO NWs are 0.31%, 1.89%, and 2.48%, respectively. All SAZO NWs show the increasing weight/atomic percent ratio of silver when the amount of the Ag/Al co-dopant is increased. This means that the silver doping concentration of SAZO NWs depends on the silver dopant ratio of the target. Also, all SAZO NWs show a low doping concentration of silver because metal combination of combined silver and aluminum with gold particle as catalyst has remained at the top of the SAZO NW confirmed by EDX analysis.

**Table 1:** EDX spectra reveal the weight/atomic percent of Zn, Al, and Ag elements in SAZO NWs

	**1 SAZO**		**3 SAZO**		**5 SAZO**	
	**Wt.%**	**At.%**	**Wt.%**	**At.%**	**Wt.%**	**At.%**
Zn K	98.84	99.59	95.63	96.84	94.28	95.93
Al K	00.85	00.28	02.48	02.14	03.24	02.82
Ag L	00.31	00.17	01.89	01.02	02.48	01.25

In order to understand the origins of the chemical bonding, the binding energies of both related Ag and Al elements in 1, 3, and 5 at.% SAZO NWs are investigated by XPS measurement as shown in Figure 4. The binding energy of Ag_2_-Al metallic bond in 1 at.% SAZO NWs is observed at 368.3 eV. In other words, the chemical bonding of Ag-O (with the role of an acceptor) is not observed in 1 at.% SAZO NWs as shown in Figure 4a. The silver ions are substituted in the Zn site when the Ag/Al co-dopants are successfully doped in ZnO NWs. However, the XPS data of 1 at.% SAZO NWs show only a Ag_2_-Al metallic bond which acts as an interstitial defect in ZnO NWs. It means that the doping condition of 1 at.% Ag/Al co-dopants could not act as a desirable acceptor in ZnO-based NWs. However, the XPS data of 3 and 5 at.% SAZO NWs show Zn, O, and Ag orbital as shown in Figure 4b,c, respectively. A sharp, strong peak which originated from the Ag chemical bonding peak (Ag 3d5/2) of the SAZO NWs is observed at 369.2 and 369.1 eV for 3 and 5 at.% SAZO NWs, respectively, as shown in the inset of Figure 4b,c. Also, the binding energies of both 369.2 and 369.1 eV are close to Ag 3d5/2 of the Ag-O bond. The Ag 3d5/2 peaks of 3 and 5 at.% SAZO NWs show a very sharp and high intensity which means that Ag dopants are successfully doped into the ZnO structure. From these results, the doping concentrations of 3 and 5 at.% Ag/Al co-dopants are best conditions to make p-type ZnO NWs.

**Figure 5:**
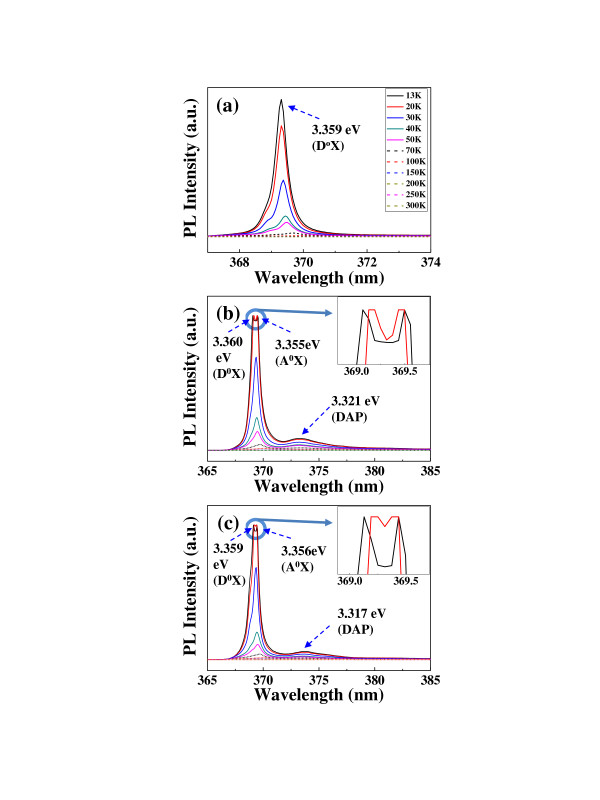
**Temperature-dependent PL spectra from (a) 1, (b) 3, and (c) 5 at.% SAZO NWs.** The temperature dependence of PL of the SAZO provides the reference for the PL analysis of the co-doped ZnO NWs, in which dominant peaks of the exciton bound to the neutral acceptor are clearly found at 3.355 and 3.356 eV for 3 and 5 at.% SAZO NWs, respectively. It demonstrates the successful Ag doping into the ZnO structure with both 3 and 5 at.% Ag/Al co-doping conditions.

Figure 5 shows the temperature-dependent PL spectra of various SAZO NWs. With increasing temperature from 13 to 300 K, the exciton peaks of various SAZO NWs are screened and shifted due to the phonon vibration and the thermal release of electrons from the shallow level [24]. Sharp, strong peaks which originated from the near-band-edge (NBE) emission of all SAZO NWs are observed at around 369 nm. In the case of 1 at.% SAZO NW, it shows only one strong peak at the 3.359 eV which is the exciton bound to the neutral donor (D^0^X) as shown in Figure 5a. This means that the condition of 1 at.% Ag/Al concentration could not be functionalized as a p-type dopant in the ZnO NWs. In other words, 1 at.% SAZO NW is an n-type nanostructure. Therefore, 1 at.% SAZO NWs do not show acceptor-related peaks such as A^0^X and donor-acceptor pair (DAP). However, in the cases of 3 and 5 at.% SAZO NWs, they show two kinds of peaks at around 3.36 eV including D^0^X and A^0^X peaks at low temperature. This means that the optical property of 3 and 5 at.% SAZO NWs is changed from an n-type semiconductor to a p-type semiconductor. Hwang et al. revealed that the peak around 3.355 eV is A^0^X-related by a phosphorous dopant [25]. Therefore, the observed peaks at 3.355 (Figure 5b) and 3.356 eV (Figure 5c) should be related with A^0^X in 3 and 5 at.% SAZO NWs, respectively. So, the Ag dopants of 3 and 5 at.% Ag/Al co-doping concentrations can easily act as the p-type acceptor. In other words, optimized 3 and 5 at.% Ag/Al co-doping concentrations are good conditions to make optically p-type ZnO NWs as confirmed by EDX, XPS, and PL measurements. As the temperature decreases, the NBE peaks are shifted from a long wavelength to a short wavelength due to the bandgap broadening effect [26-28]. Other peaks of 3 and 5 at.% SAZO NWs located at 3.321 and 3.317 eV appeared at low temperature as shown in Figure 5b,c. However, 1 at.% SAZO NWs do not show weak emission peaks at around 3.31 eV. According to the report of Zhang et al., the DAP peak of ZnO-related NWs at around 3.31 eV is observed in the 3 and 5 at.% SAZO NWs [29]. Therefore, the weak emission peaks in the 3 and 5 at.% SAZO NWs at around 3.31 eV are the DAP peaks.

**Figure 6:**
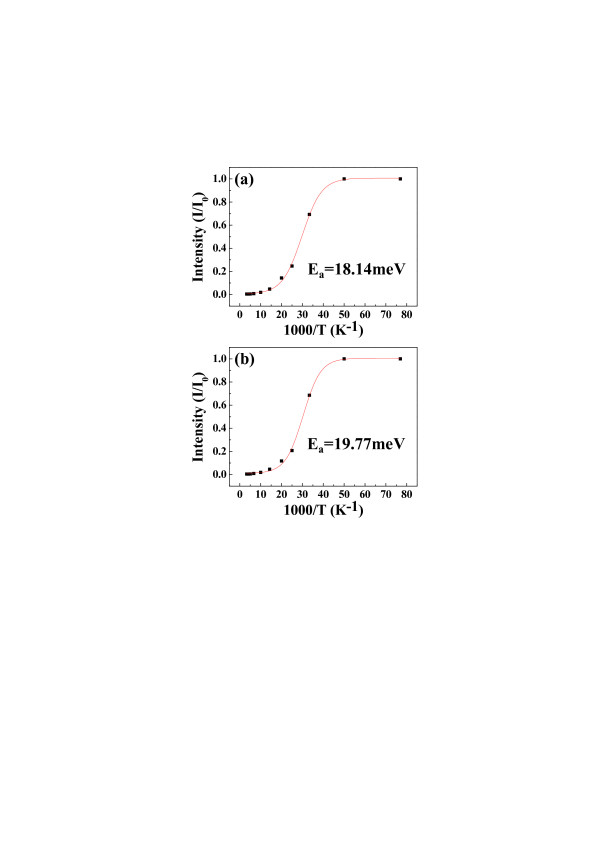
**The Arrhenius plots of the exciton bound to the neutral acceptor's PL intensity.** (**a**) 3 and (**b**) 5 at.% SAZO NWs. Resultant activation energies of the A^0^X formation are calculated to be about 18.14 and 19.77 meV for 3 and 5 at.% SAZO NWs, respectively.

Figure 6 shows the Arrhenius plots of A^0^X peaks on 3 and 5 at.% SAZO NWs. The *E*_a_ of 1 at.% SAZO NWs could not be calculated because 1 at.% SAZO NWs have only D^0^X peak. This result demonstrates that the A^0^X peaks of various SAZO NWs are very sensitive depending on the concentration of Ag/Al co-dopants. From the Arrhenius equation, the *E*_a_ can be expressed as [30]:

(1) $$I=\frac{A}{\left(1+B\text{ exp}\left(\frac{-E_{\text{a}}}{kT}\right)\right)}\text{,}$$

where *A* and *B* are the scaling factors, *E*_a_ is the activation energy, and *k* is Boltzmann's constant. Resultant activation energies of the A^0^X peaks are calculated to be about 18.14 and 19.77 meV for 3 and 5 at.% SAZO NWs, respectively. The activation energies of 3 and 5 at.% SAZO NWs show lower values compared with the activation energy of single Ag-doped ZnO NW which is higher than 25 meV in this study [31]. In other words, 3 and 5 at.% Ag/Al concentrations easily help to substitute Ag dopants in Zn sites. Consequently, 3 and 5 at.% Ag/Al co-doping methods are good conditions to make optically p-type ZnO NWs.

## Conclusions

In summary, we have synthesized various silver/aluminum-co-doped ZnO NWs on sapphire substrates by hot-walled pulsed laser deposition with Au catalysts. The XPS data of 1 at.% SAZO NWs show Ag_2_-Al metallic bond. This means that this condition generates Ag-Al metal compound which acts as an interstitial defect in ZnO NWs. Therefore, 1 at.% SAZO NWs have only one peak at 3.359 eV which originated from the D^0^X. However, new peaks of 3 and 5 at.% SAZO NWs located at 3.355 and 3.356 eV originated from the exciton bound to neutral acceptors. Also, the activation energies of 3 and 5 at.% SAZO NWs show lower values compared with the *E*_a_ of single Ag-doped ZnO NWs. We can conclude that 3 and 5 at.% Ag/Al co-doping methods facilitate to make optically p-type ZnO NWs.

## Competing interests

The authors declare that they have no competing interests.

## Authors’ contributions

KK conceived the study, conducted the experiments, performed the characterization, analyzed the data, interpreted the results, and wrote the manuscript. DHL and GEJ helped in the technical support for experiments and characterization. SYL and JSK designed the experiments, supervised the study, and corrected the manuscript. All authors read and approved the final manuscript.
